# Hidden Impacts of Peritoneal Dialysis on the Endocrine System

**DOI:** 10.3390/life15101588

**Published:** 2025-10-11

**Authors:** Hiromichi Ueno, Yoichi Ueta, Jun-ichiro Koga, Takashi Maruyama, Tetsu Miyamoto, Masaharu Kataoka

**Affiliations:** 1 Department of the Second Department of Internal Medicine, University of Occupational and Environmental Health, Kitakyushu 807-8555, Japan; hiromichi-u@med.uoeh-u.ac.jp (H.U.); j-koga@med.uoeh-u.ac.jp (J.-i.K.); mkataoka@med.uoeh-u.ac.jp (M.K.); 2 Physiology, School of Medicine, University of Occupational and Environmental Health, Kitakyushu 807-8555, Japan; yoichi@med.uoeh-u.ac.jp (Y.U.); mtaka@med.uoeh-u.ac.jp (T.M.)

**Keywords:** peritoneal dialysis, hypothalamus, biocompatibility

## Abstract

Peritoneal dialysis (PD) is a widely used renal replacement therapy in which hyperosmolar solutions are instilled into the abdominal cavity to facilitate the removal of excess water, electrolytes, and metabolic waste products. During PD treatment, homeostasis is maintained through adaptive responses of the neuroendocrine system to high glucose exposure, changes in circulating blood volume, and shifts in electrolyte balance. Clinical observations and limited experimental studies suggest that these neurohormonal dynamics may influence both the complications and therapeutic efficacy of PD. However, systematic investigations remain scarce, largely because hormonal and neural responses are highly dynamic, involve complex interactions, and are substantially influenced by individual patient characteristics. In this review, we synthesize current clinical and experimental evidence linking PD-related complications with hidden hormone dynamics, with particular emphasis on hypothalamic hormones such as arginine vasopressin. We also discuss how the biocompatibility of PD solutions—traditionally assessed by their effects on peritoneal mesothelial cells—could be reconsidered when neuroendocrine aspects are taken into account. We propose that integrating both clinical insights and emerging basic research will provide a more comprehensive understanding of neuroendocrine regulation in PD and may contribute to the development of novel therapeutic strategies.

## 1. Introduction

Patients with end-stage renal disease (ESRD) typically present with organ dysfunction and cellular injury resulting from arteriosclerosis related to the primary disease and/or the accumulation of uremic toxins. In addition to these well-recognized complications, many patients with ESRD also develop profound endocrine abnormalities [1,2]. The pathogenesis of these disorders is multifactorial; reduced hormone secretion due to renal dysfunction, compensatory hypersecretion aimed at correcting metabolic derangements, direct effects of uremic toxins on endocrine organs, and altered hormone metabolism associated with impaired renal clearance have all been implicated. Peritoneal dialysis (PD) is a standard renal replacement therapy that removes excess water, electrolytes, and metabolic waste products by instilling a hyperosmolar solution (peritoneal dialysis fluid, PDF) into the abdominal cavity for extended periods. While PD and other renal replacement therapies are effective in controlling fluid balance, uremic toxins, and mineral metabolism, endocrine disturbances are not typically primary treatment targets. Moreover, fluctuations in plasma osmolality, circulating blood volume, glucose, and mineral concentrations during PD may themselves contribute to the onset or exacerbation of endocrine disorders. This review highlights the often-overlooked hormonal dynamics underlying major complications in patients receiving PD. We argue that a better understanding of these interactions will be essential for optimizing PD therapy, guiding the development of next-generation PDF and adjunctive pharmacological strategies, and improving the selection and use of conventional solutions.

## 2. Fluid Retention

Fluid retention is one of the primary indications for initiating dialysis in patients with ESRD. Even after the initiation of PD, achieving adequate fluid control remains challenging, and many patients continue to exhibit persistent fluid overload [3]. Fluid retention is a serious complication that limits the long-term sustainability of PD and has been identified as a risk factor for adverse outcomes, including hypertension, cardiac hypertrophy, peritonitis, and mortality [4,5]. The main mechanisms of fluid retention in PD are thought to be inadequate overall water removal, which results from both reduced ultrafiltration due to peritoneal dysfunction and declining urinary output associated with loss of residual renal function. Repeated instillation of PDF and long-term intraperitoneal exposure cause physical stress, while non-physiological components of the solution exert chemical stress. Together, these stimuli induce detachment and shedding of peritoneal mesothelial cells, thickening and fibrosis of the submesothelium, and progressive angiogenesis, ultimately leading to peritoneal dysfunction and increased membrane permeability. Under these conditions, glucose reabsorption is enhanced, diminishing the osmotic gradient and thereby reducing ultrafiltration efficiency [6,7]. In addition to impaired fluid removal capacity caused by both peritoneal dysfunction and loss of residual renal excretion, we focus here on the involvement of hidden hormonal dynamics as a novel contributor to fluid retention in PD patients. The endocrine system plays a central role in maintaining homeostasis in response to external stimuli. During PD, neuroendocrine responses may act antagonistically to changes such as reduced circulating blood volume and increased plasma osmolality induced by hyperosmolar solutions.

Arginine vasopressin (AVP) is a key hormone regulating water and electrolyte homeostasis. It is synthesized in distinct hypothalamic nuclei with specialized functions: magnocellular neurons in the paraventricular (PVN) and supraoptic (SON) nuclei produce AVP primarily for systemic fluid regulation; parvocellular PVN neurons generate AVP that modulates hypothalamic–pituitary–adrenal axis activity; and the suprachiasmatic nucleus produces AVP involved in circadian rhythm control. Magnocellular AVP neurons are stimulated by increased plasma osmolality and decreased circulating blood volume, leading to rapid release of AVP from the posterior pituitary. AVP then acts on V2 receptors in the renal collecting duct to promote water reabsorption (antidiuresis). While this mechanism is essential for maintaining homeostasis, sustained upregulation of AVP synthesis and secretion can counteract fluid removal during PD and contribute to fluid retention. PD treatment chronically exposes the body to hyperosmolar conditions and/or intravascular hypovolemia, as hyperosmolar PDF is instilled into the peritoneal cavity and water is removed along the osmotic gradient. In response, AVP synthesis and secretion may be upregulated to enhance renal water reabsorption and maintain homeostasis. A trade-off between ultrafiltration and urine volume has been reported in PD patients [8]. This phenomenon can be explained by the compensatory upregulation of AVP in response to intravascular volume depletion and hyperosmolality, which counteracts the fluid removal achieved by PD. Notably, one study reported that residual renal function does not necessarily correlate with fluid retention [9]. Given that AVP exerts stronger effects when renal function is preserved, this observation may support a central role for AVP in the pathophysiology of fluid overload during PD. AVP may play an important role in fluid retention in many PD patients, and its dynamics should be considered when formulating fluid management strategies. However, accurately assessing changes in AVP synthesis and secretion under physiological conditions remains challenging. One reason is the intrinsic properties of AVP: it is highly sensitive to changes in plasma osmolality and circulating blood volume, and it is rapidly degraded, with a short plasma half-life of 5–20 min [10]. Although these features enable tight regulation of fluid and electrolyte balance, they complicate the assessment of AVP kinetics during PD, as instantaneous plasma concentrations do not necessarily reflect hypothalamic synthesis or posterior pituitary release.

**Figure 1:**
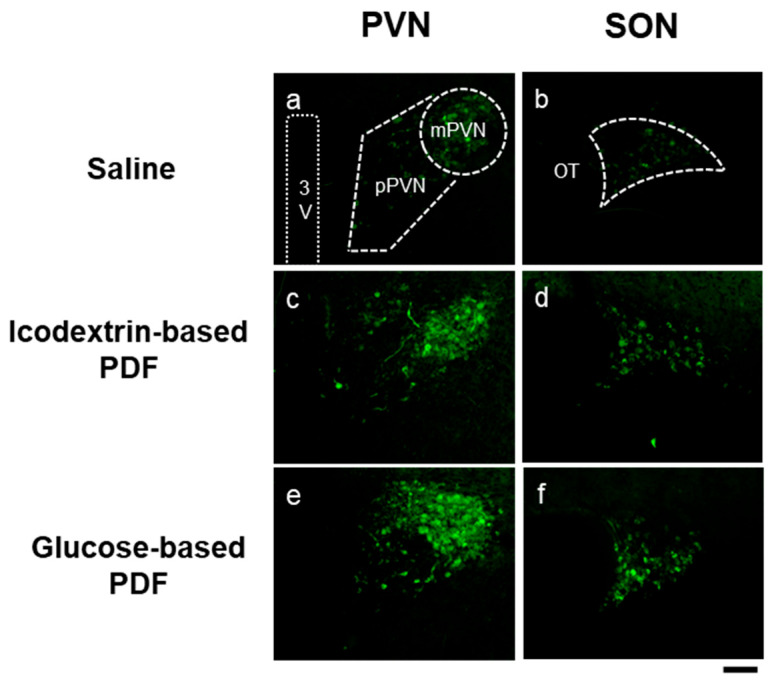
Representative images of arginine vasopressin-enhanced green fluorescent protein (AVP–eGFP) fluorescence (green cytoplasmic signal) after administration of saline (**a**,**b**), icodextrin-based peritoneal dialysis fluid (I-PDF; Extraneal, Baxter Healthcare, Round Lake, IL, USA) (**c**,**d**), and glucose-based peritoneal dialysis fluid (G-PDF; Reguneal, Baxter Healthcare, Round Lake, IL, USA) (**e**,**f**) in the paraventricular nucleus (PVN; **a**,**c**,**e**) and supraoptic nucleus (SON; **b**,**d**,**f**) at 3 h after administration. Black scale bar = 100 μm. Abbreviations: AVP, arginine vasopressin; eGFP, enhanced green fluorescent protein; I-PDF, icodextrin-based peritoneal dialysis fluid; G-PDF, glucose-based peritoneal dialysis fluid; PVN, paraventricular nucleus; SON, supraoptic nucleus; OT, optic tract; 3V, third ventricle; mPVN, magnocellular division of the PVN; pPVN, parvocellular division of the PVN. Reproduced from Ueno et al., Peritoneal Dialysis International (2025) [Online ahead of print], with permission from SAGE Publications [14].

To overcome these limitations, we employed transgenic rats expressing enhanced green fluorescent protein (eGFP) under the AVP promoter, which allowed us to visualize and semi-quantitatively evaluate hypothalamic AVP synthesis under various physiological conditions [11,12]. Fluorescence intensity correlates with AVP synthesis, and eGFP also serves as a marker to identify AVP neurons. Furthermore, neuronal activity in response to PDF can be assessed by examining c-Fos expression in AVP neurons and other hypothalamic nuclei [11]. Using this approach, we sought to visualize changes in hypothalamic AVP during PD. In the first stage of our experiments, two simple solutions were used as PD models: hypertonic saline (HTN) as a short-acting PDF, polyethylene glycol (PEG) as a long-acting PDF, and saline as a control. Time-dependent changes in hypothalamic AVP synthesis were evaluated over 24 h. Compared with controls, both HTN and PEG administration increased AVP synthesis in the magnocellular hypothalamic regions [13]. In the second stage, we examined the effects of clinically used PD solutions—glucose-based PDF (G-PDF; Reguneal, Baxter Healthcare, Round Lake, IL, USA) and icodextrin-based PDF (I-PDF; Extraneal, Baxter Healthcare)—on AVP in both clinical and experimental settings. In PD patients, plasma AVP concentrations tended to be higher than baseline values. Although no significant difference was observed compared with ESRD patients prior to dialysis initiation, this may reflect the opposing influences of toxin removal by PD and volume load from fluid reabsorption, which can suppress AVP upregulation. As noted, plasma AVP levels are highly labile and may not reliably reflect sustained hypothalamic synthesis. In parallel animal experiments, administration of both G-PDF and I-PDF to AVP-eGFP rats increased hypothalamic AVP biosynthesis (Figure 1). Clinically, we also observed a correlation between AVP and urinary osmolality in PD patients who retained urine output, indicating that AVP function is preserved during PD treatment [14]. Consistently, although large-scale clinical trials are lacking, previous reports have shown that tolvaptan, a V2 receptor antagonist, increases urine volume in PD patients [15]. In the third stage of this research, we aim to develop novel PD solutions that do not induce AVP synthesis and to evaluate their therapeutic potential and biological effects. We believe that this series of experiments provides new insights into peritoneal dialysis treatment; however, several limitations of the experimental design should be acknowledged. First, the rats used in these experiments were young and had normal renal function. This condition likely precluded fluid retention, uremia, electrolyte imbalances, or the accumulation of other metabolites that could influence endocrine function. In addition, the peritoneal dialysis solution was administered only once; repeated or long-term administration might produce markedly different outcomes. Despite these limitations, we consider this work to be an important step toward elucidating the potential impact of peritoneal dialysis solutions on the endocrine and nervous systems—an area that has not been systematically investigated to date.

**Figure 2:**
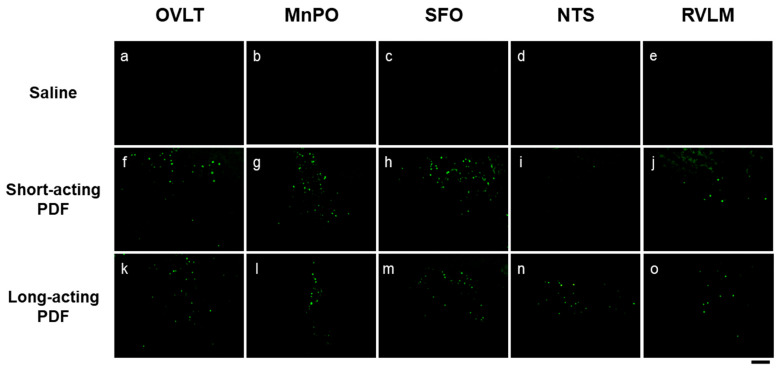
Representative images of fluorescent immunohistochemistry (FIHC) for Fos expression (green, round-shaped nuclei) after administration of saline (**a**–**e**), short-acting peritoneal dialysis fluid (PDF) (hypertonic saline, HTN; **f**–**j**), or long-acting PDF (polyethylene glycol, PEG; **k**–**o**). Images show the organum vasculosum of the lamina terminalis (OVLT; **a**,**f**,**k**), median preoptic nucleus (MnPO; **b**,**g**,**l**), subfornical organ (SFO; **c**,**h**,**m**), nucleus tractus solitarius (NTS; **d**,**i**,**n**), and rostral ventrolateral medulla (RVLM; **e**,**j**,**o**). Black scale bar = 100 μm. Abbreviations: FIHC, fluorescent immunohistochemistry; PDF, peritoneal dialysis fluid; PEG, polyethylene glycol; OVLT, organum vasculosum of the lamina terminalis; MnPO, median preoptic nucleus; SFO, subfornical organ; NTS, nucleus tractus solitarius; RVLM, rostral ventrolateral medulla. Reproduced from Ueno et al., Physiological Reports (2020) under the terms of the Creative Commons Attribution License (CC BY 4.0) [21].

The pathways regulating AVP synthesis and secretion in the hypothalamus involve both the circumventricular organs (CVOs) and the autonomic nervous system. CVOs are highly vascularized but lack a blood–brain barrier, enabling them to detect increases in plasma osmolality and angiotensin II and activate hypothalamic AVP neurons. In addition, atrial volume receptors and arterial baroreceptors sense decreases in circulating blood volume and promote AVP release via sympathetic activation. In our simple PD models, administration of short-acting PDF (HTN) and long-acting PDF (PEG) induced c-Fos expression, a marker of neuronal activity, in representative circumventricular organs (CVOs)—including the organum vasculosum of the lamina terminalis (OVLT), median preoptic nucleus (MnPO), and subfornical organ (SFO)—as well as in autonomic control nuclei such as the nucleus tractus solitarius (NTS) and rostral ventrolateral medulla (RVLM) (Figure 2) These findings suggest that short-acting PDFs stimulate CVOs through hyperosmolality, whereas long-acting PDFs activate sympathetic outflow through reduced circulating volume. Clinically, PD patients more frequently complain of dry mouth than those receiving hemodialysis [16]. This symptom may induce drinking behavior and contribute to fluid retention. Although the mechanism is not fully clarified, hyperosmolality during PD is thought to be a major cause. Our experimental results demonstrated activation of CVOs, the central regulators of thirst, in PD models. It is therefore plausible that stimulation of CVOs by PDF contributes to enhanced drinking behavior and fluid overload. Fos expression is a useful marker for assessing central nervous system activity during the acute phase; however, because it primarily reflects only the presence or absence of neuronal excitation, it has inherent limitations in quantification and temporal resolution. In addition, the activation of inhibitory neurons may suppress downstream neural activity or alter endocrine function, so such data must be interpreted with caution. Moreover, the findings presented here were obtained using a simple PDF in our experiment. In future studies, we plan to examine the effects of clinically used PDF and incorporate behavioral analyses to achieve a more comprehensive understanding. Chronic hypovolemia due to ultrafiltration or albumin leakage into the peritoneal cavity may also stimulate the renin–angiotensin–aldosterone system (RAAS). Elevated plasma renin activity has been reported in PD patients compared with hemodialysis patients, suggesting RAAS activation during PD [17,18]. Persistently elevated aldosterone may contribute to hyperkalemia and myocardial remodeling. Clinical studies have shown that RAAS inhibitors and aldosterone antagonists help preserve residual renal function and urinary output [19,20]. Since angiotensin II is known to stimulate AVP secretion, RAAS activation could represent an additional mechanism for AVP upregulation during PD.

Oxytocin (OXT), which is synthesized in the hypothalamus and secreted by the posterior pituitary, also regulates systemic fluid and electrolyte balance. OXT and AVP are genetically homologous, share similar receptors and regulatory mechanisms, and are often co-expressed. In our experiments, both PEG and HTN induced hypothalamic OXT synthesis in parallel with AVP [21]. OXT has been shown to facilitate natriuresis and salt balance through suppression of salt intake, direct renal actions, and stimulation of atrial natriuretic peptide secretion [22,23,24,25]. While OXT and AVP exert partially antagonistic physiological effects, several reports suggest that OXT can produce antidiuretic effects through the AVP V2 receptor [26,27]. Thus, the precise role of OXT in fluid regulation during PD remains unclear, and its interaction with AVP requires further study.

## 3. Autonomic Nervous System Abnormality

The autonomic nervous system (ANS) is a central mechanism for maintaining homeostasis, operating through feedback circuits within the central nervous system. Neural inputs such as renal sympathetic afferents and arterial baroreceptor reflexes converge on the NTS, while humoral factors, including circulating hormones and uremic toxins, are detected by CVOs, such as the SFO and relayed to the hypothalamic PVN. The RVLM functions as the final integration center, receiving inhibitory input from the NTS and excitatory input from the PVN, thereby determining sympathetic efferent output [28]. Sympathetic nerves innervate both afferent and efferent arterioles of the renal glomerulus, and sustained sympathetic overactivation has been associated with progression of renal dysfunction and increased proteinuria [29,30]. Heightened sympathetic activity is frequently observed in patients with chronic kidney disease (CKD) [31]. Experimental studies have further shown that renal afferent denervation prevents the rise in central noradrenaline induced by renal injury or high-salt intake [32], suggesting reciprocal interactions between renal dysfunction and sympathetic activation. Recent evidence indicates that certain uremic solutes may directly stimulate RVLM activity, promoting sympathetic excitation and thereby creating a vicious cycle that exacerbates renal impairment [33].

Clinical studies demonstrate that plasma norepinephrine concentrations, a marker of sympathetic activity, are elevated in patients undergoing PD. Interestingly, catecholamine levels in PD patients are lower than in predialysis ESRD patients but higher than in those receiving hemodialysis [18,34], which may reflect differences in toxin clearance efficiency between dialysis modalities. These findings suggest that PD modifies ANS activity both by alleviating uremia and by introducing additional physiological stresses. Inputs from fluid overload and renal pelvic congestion in ESRD, together with uremic toxins, are transmitted via renal afferents to the CNS, where they suppress sympathetic output through inhibitory pathways in the NTS. However, the removal of fluid by PD may relieve this inhibitory tone, resulting in increased sympathetic drive.

As described in the Fluid retention section, we visualized neuronal activation in animals subjected to a simple PD model by examining the expression of Fos protein, a marker of neuronal activity. In this model, administration of PDF induced Fos expression in the NTS and RVLM, both of which are key nuclei regulating ANS activity [21]. Because these animals lacked renal dysfunction and uremia, the findings suggest that PDFs themselves may exert direct pharmacological effects on the ANS. Endocrine pathways may also contribute: subsets of hypothalamic AVP neurons project to the RVLM and spinal cord, providing excitatory input to sympathetic activity [35]. Indeed, increased AVP secretion has been reported to activate renal sympathetic nerves [36,37]. Taken together, these observations suggest two opposing mechanisms: while uremia alleviation by PD may suppress sympathetic activity, PDF administration could directly or indirectly enhance sympathetic outflow via AVP upregulation.

**Figure 3:**
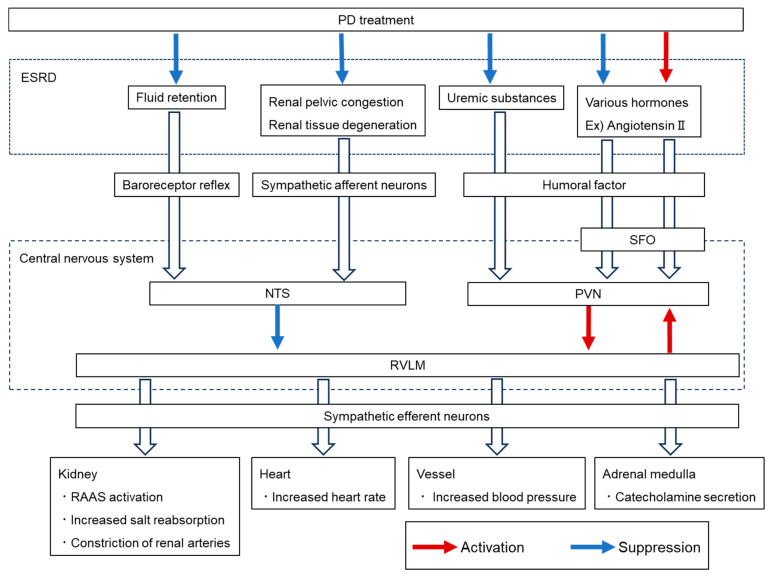
Schematic representation of the effects of end-stage renal disease (ESRD)-related abnormalities and peritoneal dialysis (PD) treatment on autonomic nervous system (ANS) activity. Inputs from uremic toxins, fluid retention, angiotensin II, and renal injury-related signals converge on circumventricular organs (CVOs) such as the subfornical organ (SFO) and on renal sympathetic afferents projecting to the nucleus tractus solitarius (NTS). Excitatory input from the paraventricular nucleus (PVN) and inhibitory input from the NTS are integrated in the rostral ventrolateral medulla (RVLM), which regulates efferent sympathetic output to the heart, kidney, and vasculature. Chronic activation contributes to increased heart rate, renin–angiotensin–aldosterone system (RAAS) activation, enhanced sodium reabsorption, renal vasoconstriction, elevated blood pressure, and catecholamine secretion from the adrenal medulla. In this figure, excitatory pathways from the central nervous are indicated in red and inhibitory pathways in blue. Abbreviations: ANS, autonomic nervous system; AVP, arginine vasopressin; CVO, circumventricular organ; SFO, subfornical organ; NTS, nucleus tractus solitarius; PVN, paraventricular nucleus; RVLM, rostral ventrolateral medulla; PD, peritoneal dialysis; RAAS, renin–angiotensin–aldosterone system.

Overall, the ANS is inherently dynamic, and it remains uncertain whether the increase in sympathetic activity during PD represents a persistent state or a transient response, especially when compared with the marked hemodynamic fluctuations observed during hemodialysis. The reciprocal regulation between endocrine and neural systems further complicates the interpretation of these mechanisms, underscoring the need for integrated clinical and basic research approaches (Figure 3).

## 4. Impaired Glucose Tolerance

Diabetes mellitus (DM) is the leading primary cause of ESRD worldwide, and many PD patients experience impaired glucose tolerance due to comorbidities and steroid use [38]. The endocrine system, particularly insulin, plays a central role in this pathophysiology. Even in the absence of DM, CKD patients develop early insulin resistance [39]. Patients with ESRD secondary to or complicated by diabetes mellitus generally exhibit advanced arteriosclerosis and are at high risk of developing cardiovascular complications; therefore, optimal glycemic control is essential. However, the peritoneal dialysis fluids currently in widespread use are predominantly high-glucose solutions, largely due to their cost-effectiveness and clinical practicality [40]. Because glucose in PDF is small in molecular size, it is readily absorbed into the systemic circulation. During continuous ambulatory PD (CAPD), the standard modality of continuous therapy, the absorbed glucose load is estimated at 100–300 g per day [41]. Although there has been concern that this chronic exposure worsens glucose tolerance, recent epidemiological studies suggest that the incidence of new-onset glucose intolerance after PD initiation is not higher than that after hemodialysis initiation [42,43]. The apparent discrepancy may be explained by the fact that renal replacement therapy itself alleviates uremia, improves insulin sensitivity, and counterbalances the effects of glucose absorbed from PDF [44].

Insulin resistance not only impairs glucose control but also disrupts protein anabolism, contributing to muscle wasting and sarcopenia. Since sarcopenia strongly affects prognosis and quality of life in PD patients, clarifying the mechanisms of insulin resistance and controlling blood glucose are important for both acute complication prevention and long-term outcomes. The major mechanisms of insulin resistance in CKD are thought to involve pro-inflammatory cytokines such as tumor necrosis factor-α (TNF-α), interleukin-6 (IL-6), and interferon-γ (IFN-γ), which inhibit phosphorylation of the insulin receptor and insulin receptor substrate-1 (IRS-1) [45]. RAAS activation, particularly increased angiotensin II, also promotes IL-6 and amyloid A production and decreases IRS-1, thereby impairing insulin action [46]. In ESRD, uremic toxins such as p-cresyl sulfate (PCS), a metabolite of tyrosine, have been implicated in insulin resistance [47]. Moreover, elevated cytokines and oxidative stress have been reported in PD patients, suggesting that these factors contribute to impaired insulin signaling [48,49].

The metabolic impact of chronic intraperitoneal glucose exposure varies widely among patients, depending not only on insulin sensitivity but also on peritoneal membrane characteristics [50]. In addition to insulin-mediated mechanisms, hidden endocrine responses triggered by PDF may also influence glycemic control. Cortisol, a key hyperglycemic hormone, is elevated in PD patients [17]. Adrenal responsiveness to ACTH appears preserved, and abnormal cortisol levels may be mediated centrally [51]. Hypothalamic AVP is involved in adrenal regulation through interactions with corticotropin-releasing hormone (CRH) in the parvocellular PVN, jointly stimulating ACTH secretion in the anterior pituitary. Our studies demonstrated that intraperitoneal PDF administration activates not only the magnocellular but also the parvocellular hypothalamus. Specifically, PEG-based solutions significantly increased AVP synthesis in the parvocellular region in experimental models [13], and clinical G-PDF also activated parvocellular AVP neurons. Contrary to our expectations, I-PDF had a weaker effect on parvocellular AVP synthesis compared with G-PDF [14].

Furthermore, hypothalamic AVP and OXT have been shown to suppress feeding behavior via vagal afferent pathways [52]. In a simple PD model, both AVP and OXT synthesis were upregulated [21]. Such changes in feeding behavior may contribute to glycemic variability during PD. In addition, glucagon levels—a major counter-regulatory hormone—are elevated in ESRD patients with uremia and rise even further in those on PD [53]. Collectively, these findings suggest that glucose intolerance in PD patients is influenced not only by glucose absorption and insulin resistance but also by complex neuroendocrine responses involving cortisol, AVP, OXT, and glucagon.

## 5. Bone and Mineral Disorders

Chronic kidney disease–mineral and bone disorder (CKD-MBD) is defined by abnormalities in calcium, phosphorus, parathyroid hormone (PTH), and vitamin D metabolism, along with bone pathology and soft tissue calcification. These alterations substantially affect survival and cardiovascular morbidity in CKD patients [54]. The KDIGO guidelines recommend treatment strategies aimed at preventing vascular and skeletal complications across the stages of CKD and provide target ranges for phosphate, calcium, and PTH in dialysis patients [55]. In PD patients, as in HD patients, serum phosphorus concentrations correlate with bone mineral density, vascular calcification, and survival outcomes [56,57].

The main difference between HD and PD lies in the duration and continuity of treatment. HD is performed intermittently (typically 4–6 h, three times per week), whereas PD provides continuous therapy (6–24 h per day). Consequently, in patients undergoing PD, serum calcium, phosphorus, and PTH levels tend to remain relatively stable regardless of the timing of sampling. This differs substantially from HD, in which these parameters fluctuate markedly with each dialysis session, even though predialysis values are generally regarded as baseline for both modalities. Target ranges for calcium, phosphorus, and PTH are set at similar levels for PD and HD patients. However, even if pretreatment values are comparable between the two groups, differences in treatment characteristics may result in higher daily average values of serum parameters in PD patients compared with HD patients. Therefore, some researchers suggest that earlier intervention may be warranted in PD patients, even when these parameters are within the upper normal range [58]. In CAPD, the PTH clearance rate is approximately 1.5 mL/min, corresponding to removal of 13.6% of extracellular hormone content per day—greater than that achieved by HD [59]. Some studies report a decline in PTH after PD initiation, whereas others show progressive increases, leaving conclusions uncertain [60,61]. Importantly, PTH levels are determined not only by production and dialysis clearance, but also by residual renal function, dietary intake, electrolyte balance, inflammation, and circadian variation [6].

Fibroblast growth factor-23 (FGF-23), produced mainly by osteocytes, regulates phosphorus and vitamin D metabolism by inhibiting phosphate reabsorption and suppressing renal 1,25-dihydroxyvitamin D synthesis. FGF-23 also influences the musculoskeletal system. Serum FGF-23 levels are elevated early in CKD and strongly correlate with phosphorus levels in both predialysis and dialysis patients [59]. Among PD patients, FGF-23 correlates with serum phosphorus, residual renal function, and PD duration [62]. Nonetheless, FGF-23 concentrations are consistently higher in PD than HD patients, independent of calcium and phosphorus levels [63]. Elevated FGF-23 has been linked to cardiovascular events and infections, and monitoring may help identify high-risk groups, although it remains unclear whether lowering FGF-23 improves outcomes.

Calcitonin, secreted by the thyroid, promotes phosphate excretion and inhibits osteoclastic bone resorption. Plasma calcitonin levels are elevated in both PD and HD patients, with approximately 80% of PD patients showing high concentrations. However, compared with HD patients, PD patients tend to have lower calcium and calcitonin levels, and calcitonin correlates with blood calcium [64]. This discrepancy may be related to impaired renal clearance combined with higher peritoneal excretion of calcitonin (3–4 mL/min) in PD compared with HD [64].

OXT, which we found to be upregulated during PD treatment, also influences musculoskeletal metabolism. Experimental studies show that OXT suppresses adipogenesis in adipose-derived stem cells and promotes osteogenesis in mesenchymal stem cells [21]. In murine models of menopausal osteoporosis, OXT administration reversed bone loss and reduced marrow adiposity [26]. OXT further contributes to muscle regeneration by stimulating aged muscle stem cells via the MAPK/ERK pathway, although circulating levels decline with age [65]. Since sarcopenia and bone mineral loss are poor prognostic factors in renal failure, modulation of hypothalamic OXT by PDF may represent a novel therapeutic target to mitigate these complications.

Conversely, activation of the hypothalamic–pituitary–adrenal (HPA) axis during PD treatment could have detrimental skeletal effects [13,14]. Corticosteroids, including cortisol in humans and corticosterone in rodents, downstream of parvocellular AVP neurons, are well known to induce osteoporosis. Glucocorticoids inhibit osteoblast formation and proliferation, increase the RANKL/OPG ratio to enhance osteoclast activity, and prevent osteoclast apoptosis, thereby promoting bone resorption [66,67]. Taken together, activation of the HPA axis by PDF may contribute to bone mineral density loss. Intriguingly, AVP and OXT, which share homologous genes and regulatory mechanisms, exert opposing effects on bone metabolism—highlighting the complex neuroendocrine regulation of skeletal health in PD.

## 6. Renal Anemia

The prevalence of renal anemia increases with the progression of kidney dysfunction. Inadequate anemia control is associated with reduced quality of life, cardiovascular complications, hospitalization, cognitive decline, and mortality. The pathophysiology of renal anemia involves decreased erythropoietin production, iron deficiency, and shortened red blood cell lifespan. Erythropoietin replacement therapy is the first-line treatment; however, anemia appears less frequent and less severe in PD patients compared with HD patients, with correspondingly lower use of erythropoietin-stimulating agents (71.4% vs. 86.9%) [68]. Switching from HD to PD has been associated with increased red blood cell counts and decreased serum ferritin levels, possibly reflecting better preservation of residual renal function, avoidance of bleeding related to vascular access and heparin, and reduced inflammation [69]. Interestingly, serum erythropoietin concentrations increase significantly after switching to PD compared with HD, a finding not fully explained by these mechanisms [70]. Moreover, combined PD and HD therapy has been shown to improve erythropoietin-resistant anemia [71], suggesting that dialysis efficiency, rather than modality alone, may be more critical for anemia management.

Beyond erythropoietin, other hormones also influence hematopoiesis. PTH, for example, promotes anemia by reducing endogenous erythropoietin production and shortening red blood cell survival [72]. PTH can also impair hematopoiesis indirectly by inducing bone marrow fibrosis [73]. Clinical studies show that parathyroidectomy in ESRD patients with secondary hyperparathyroidism improves anemia, erythropoietin production, and responsiveness [74]. Similarly, fibroblast growth factor-23 (FGF23), a key regulator in CKD-MBD, has been implicated in renal anemia. FGF23 may aggravate anemia through direct mechanisms, such as suppressing erythropoietin secretion and inducing red blood cell apoptosis, and indirect mechanisms, including effects on iron metabolism via inflammatory cytokine induction [75,76]. Inhibition of FGF23 signaling in a mouse model of renal failure improved anemia by increasing erythropoietin levels, red blood cell counts, and bone marrow progenitors [77]. These findings suggest that FGF23 could represent a novel therapeutic target for renal anemia in patients with PD [78].

Finally, the hypothalamic hormone AVP—discussed throughout this review—may also influence erythropoiesis. Basic studies indicate that AVP promotes proliferation and differentiation of red blood cell precursors independent of erythropoietin [79,80]. Clinically, anemia is more prevalent in patients with central diabetes insipidus, in whom hypothalamic AVP synthesis is deficient, although direct evidence linking AVP to renal anemia in PD is lacking [79]. We have reported that PDF can stimulate hypothalamic AVP synthesis. While this review has highlighted the potential role of AVP as a contributor to PD complications, it is also possible that AVP upregulation exerts beneficial effects on anemia in PD patients. This duality underscores the complexity of neuroendocrine dynamics: endocrine changes associated with PD cannot be judged as purely beneficial or harmful when evaluated in isolation, but rather must be understood within the broader physiological context.

## 7. Limitations and Future Perspectives

This review has highlighted important findings from various clinical and animal studies regarding the endocrine effects of peritoneal dialysis; however, direct translation to human patients remains limited. As discussed, the endocrine system comprises complex regulatory circuits involving the nervous system and is strongly influenced by individual factors such as underlying diseases and dialysis conditions. Moreover, recent reviews have pointed out that endocrine-disrupting chemicals (EDCs), which can interfere with hormonal regulation and exert adverse health effects, are not efficiently removed by renal replacement therapies [81]. Clinical studies validating these findings are still scarce, and further prospective investigations in PD populations are warranted.

In addition, the interactions between novel PD solutions and neuroendocrine regulation have not yet been systematically examined. Several limitations in experimental design should also be acknowledged. For instance, commonly used rodent PD models do not fully reproduce end-stage renal failure, and their metabolic and endocrine responses may differ from those of human PD patients. These limitations underscore the need for caution when extrapolating preclinical results. Future research should aim to bridge these translational gaps through integrated clinical and experimental approaches, ultimately advancing our understanding of the endocrine consequences of PD therapy.

## 8. Conclusions

**Figure 4:**
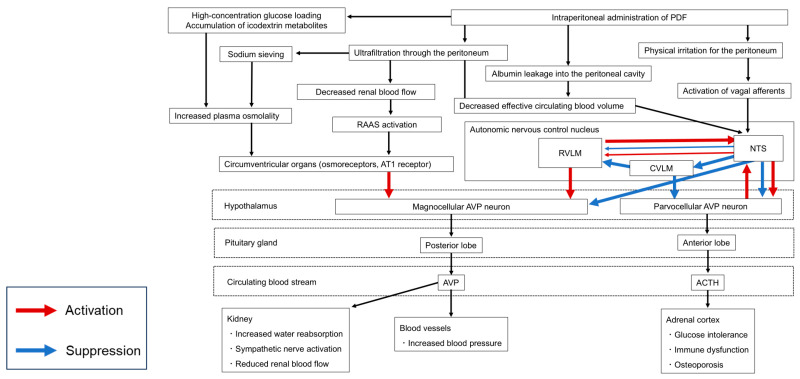
Schematic representation of hypothalamic arginine vasopressin (AVP)-mediated biological responses in patients after intraperitoneal administration of peritoneal dialysis fluid (PDF). Magnocellular AVP neurons in the paraventricular nucleus (PVN) and supraoptic nucleus (SON) are stimulated by increased plasma osmolality or decreased circulating volume and secrete AVP from the posterior pituitary. Circulating AVP then acts on V1a receptors in vascular smooth muscle, promoting vasoconstriction and elevating blood pressure. In contrast, parvocellular AVP neurons in the PVN regulate the hypothalamic–pituitary–adrenal (HPA) axis and contribute to the control of corticosterone/cortisol secretion. Together, these pathways highlight the complex involvement of AVP dynamics in the pathophysiology of PD-related complications. In this figure, excitatory pathways projecting to the hypothalamus are indicated in red and inhibitory pathways in blue.

In this review, we have summarized the hidden hormone dynamics associated with complications of PD, focusing in particular on hypothalamic AVP, a key integrator of endocrine and neural regulation. We also summarized and discussed the involvement of the endocrine system in glucose intolerance, CKD–MBD, and renal anemia, which are major complications associated with PD (Table 1). While AVP is best known for its role in water reabsorption via the renal collecting duct, our discussion highlighted that PD-induced upregulation of magnocellular AVP may contribute to fluid retention, whereas activation of parvocellular AVP neurons can stimulate the hypothalamic–pituitary–adrenal axis, potentially leading to impaired glucose tolerance. Corticosteroids downstream of this pathway also influence immune function and may contribute to PD-associated infections. Moreover, AVP regulates autonomic nervous activity, blood pressure, and bone metabolism (Figure 4). These diverse and fluctuating actions are further modulated by patient-specific factors, including underlying disease, complications, treatment regimen, and PD duration, making prediction and interpretation of AVP dynamics extremely challenging. Understanding the neuroendocrine effects of PD is therefore crucial for the development of therapeutic strategies. End-organ-targeted pharmacological interventions, such as tolvaptan, have demonstrated benefit against AVP-mediated fluid retention [15], but remain insufficient to address the full spectrum of PD-related complications linked to AVP, including hypertension and immune dysregulation. A more comprehensive approach may involve limiting PD-induced stimulation of AVP synthesis and secretion itself.

Non-physiological components contained in PDF—such as high concentrations of glucose, glucose degradation products (GDPs), high osmotic pressure, low pH, lactic acid, and plasticizers—have long been recognized as factors that cause peritoneal deterioration, leading to ultrafiltration failure and peritoneal fibrosis [7]. Consequently, “highly biocompatible” PDFs have traditionally been defined by their minimal cytotoxicity toward peritoneal mesothelial cells. However, we propose that this definition should be broadened: a truly biocompatible PDF should minimize adverse effects not only on the peritoneal membrane itself but also on systemic physiology, including the neuroendocrine system. We hope that the perspectives outlined in this review will encourage further clinical and experimental investigations and ultimately contribute to prolonging the period during which PD can be performed safely and effectively.

## Figures and Tables

**Table 1:** Summary of previous reports on hormonal factors associated with complications during peritoneal dialysis (PD), including impaired glucose tolerance, chronic kidney disease–mineral and bone disorder (CKD-MBD), and renal anemia.

Associated Complication	Hormone	Description	Key Reference
**Impaired Glucose Tolerance**	Insulin	· In CKD patients, insulin resistance develops early, irrespective of diabetes status, but tends to improve after initiation of PD.	[44]
	Cortisol	· Clinical studies have shown elevated plasma cortisol levels in patients undergoing PD, and this increase appears to be driven primarily by central rather than peripheral mechanisms.	[17,53]
		· Intraperitoneal retention of PDF may activate parvocellular AVP neurons in the hypothalamus, a key site controlling corticosterone synthesis.	[13,14]
	Glucagon	· Plasma glucagon levels are elevated in ESRD patients and are even higher in those on PD.	[53]
**CKD-MBD**	PTH	· No clear consensus exists regarding changes in plasma PTH levels in PD patients.	[60,61]
		· Plasma FGF-23 levels are higher in PD patients than in HD patients	[63]
	Calcitonin	· Plasma calcitonin levels are elevated in PD patients, but remain lower than in HD patients.	[64]
	Oxytocin	PDF administration may stimulate hypothalamic OXT neuronal activity and promote its synthesis	[21]
**Renal Anemia**	Erythropoietin	· Switching from HD to PD increases plasma erythropoietin levels, and switching from PD monotherapy to combined PD and HD improves erythropoietin-resistant anemia.	[70,71]
	PTH, FGF-23	· See “CKD-MBD” section	[57,58,63]

**Abbreviations**: PD, peritoneal dialysis; PDF, peritoneal dialysis fluid; AVP, arginine vasopressin; OXT, oxytocin; ESRD, end-stage renal disease; PTH, parathyroid hormone; FGF-23, fibroblast growth factor-23; I-PDF, icodextrin-based PD fluid; G-PDF, glucose-based PD fluid; PVN, paraventricular nucleus; NTS, nucleus tractus solitarius; RVLM, rostral ventrolateral medulla; ACTH, adrenocorticotropic hormone; cortisol, cortisol (glucocorticoid hormone); corticosterone, adrenal corticosteroid in rodents.

## Data Availability

The datasets presented in this article are not readily available because the results of the paper are not published elsewhere and are not linked to archived database which can be shared.

## Abbreviations

PDF, peritoneal dialysis fluid; RAAS, renin–angiotensin–aldosterone system; CVOs, circumventricular organs; AT1, angiotensin II type 1 receptor; NTS, nucleus tractus solitarius; CVLM, caudal ventrolateral medulla; RVLM, rostral ventrolateral medulla; PVN, paraventricular nucleus; AVP, arginine vasopressin; ACTH, adrenocorticotropic hormone; mPVN, magnocellular division of the PVN; pPVN, parvocellular division of the PVN; HPA, hypothalamic–pituitary–adrenal axis.
